# Inclusion of Indigenous Australians in biobanks: a step to reducing inequity in health care

**DOI:** 10.5694/mja2.50219

**Published:** 2019-06-03

**Authors:** Imogen Elsum, Callum McEwan, Emma E Kowal, Yvonne Cadet‐James, Margaret Kelaher, Lynn Woodward

**Affiliations:** ^1^ Centre for Health Policy University of Melbourne Melbourne VIC; ^2^ Alfred Deakin Centre for Citizenship and Globalisation Deakin University Melbourne VIC; ^3^ Indigenous Education and Research Centre James Cook University Townsville QLD; ^4^ Centre for Health Policy University of Melbourne Melbourne VIC; ^5^ College of Medicine and Dentistry and Australian Institute of Tropical Health and Medicine James Cook University Townsville QLD

**Keywords:** Biological specimen banks, Social determinants of health

Without improved practices and policy to guide the engagement and inclusion of Indigenous Australians in biobanks, the full health benefits provided by the genomic era will not be shared equitably

Biobanks are collections of biological specimens, with accompanying health and demographic information, stored and maintained for research purposes.^1^ Research may range from large scale population‐based longitudinal studies or more defined disease and tissue‐specific initiatives. In both observational and cohort studies, biobanks provide an invaluable resource that allows researchers to examine the complex range of factors which contribute to disease, without having to devote time to, and source funding for, the collection and storage of samples.

The translation of research based on samples from biobanks, particularly in genomic studies, relies on the biobank accurately representing the genetic variation present within a population. Despite this, minority group participation in biobanks remains low both in Australia and internationally.^2^ Recruitment to biobanks has typically shown a bias towards people who are tertiary educated, living in major cities, middle aged, English speaking and of higher socio‐economic standing.^3^ Another major influencing factor is that many minority populations, in particular Indigenous populations, have significant cultural concerns surrounding the collection, storage and use of biological specimens that negatively influence participation.^4^ Consequently, biobanks being utilised as a research tool may benefit select populations and therefore further exacerbate pre‐existing inequities in public health.

Concerns about the diversity of biobanks are not limited to ethical uncertainties regarding inclusivity. The misclassification of benign variants as pathogenic has led to genetic misdiagnoses for specific racial groups, and specific genetic variants across unique populations have been shown to affect drug metabolism, resulting in an ineffective or adverse effect.^5^ Therefore, the inclusion of minority groups within biobanks is not just an ethical obligation but a requirement for genomic advances that translate to the broader population. However, there are concerns that through our efforts to drive social inclusivity through minority participation we are losing analytical acuity, as the small sample size of these groups makes meaningful and statistically relevant inter‐population analysis challenging.^6^

## Genetic research in Indigenous health

Despite the increasingly prevalent role of genetics in health research, driven by advances in genomic technologies, we see little integration of genetic research and genetic services into Indigenous health in Australia. This lags significantly behind other developed nations such as the United States and Canada who have utilised genomic information and research in driving Indigenous health policy.^7^

Until recently, collection of biobank samples and subsequent genetic research has not been an area of focus in Indigenous health, highlighted by the lack of relevant publications in this field. Even now it generates a level of apprehension in Indigenous communities founded not only in the exploitive nature of our colonial past but uncertainties over data sharing and ownership rights.^8^, ^9^

Early population‐based studies such as the Human Genome Diversity Project in the 1990s aimed to explore genetic diversity of the human species by sequencing Indigenous populations.^10^ Labelled the “vampire project” by Australian Indigenous communities, there was no consultation with the communities, no transparency over what research the samples would be used for, and uncertainty surrounding the ownership and patenting rights of potential developments. This approach, without identifiable benefits to the communities, led to a public outcry which has had lasting impact on the perception of genetic research by Indigenous Australians.^10^

In stark contrast, genetic research which clearly addresses specific health concerns relevant to the community, and identified by the community, has been successful. Research in remote Indigenous communities in Arnhem Land focused on vulvar cancer, which had a disparately high incidence. This was embraced by the communities and continues to provide a model for successful community‐driven research.^11^

An open discourse between Indigenous and research communities is essential and is beginning to shape how Indigenous genetic health research is conducted in Australia. The National Centre for Indigenous Genomics, established in 2017, aims to produce a national Indigenous genomics resource and reference genome, for research to benefit Indigenous Australians and establish a more successful framework for ethical Indigenous genetic research. This involves continued community engagement at every stage of the research, a dynamic informed consent platform and complete transparency, with an Indigenous majority board providing oversight.^12^ An integral component of this resource will be the National Centre for Indigenous Genomics outreach program, which will provide genomic knowledge to Indigenous students and community members. Improving genetic literacy is an important step to better engaging Indigenous Australians on inclusion within biobanks and biomedical research.

## Diversity in biobanks internationally

Internationally, efforts towards achieving proportional diversity in biobanks have been driven by legislative obligation, by setting guidelines for minority recruitment from the onset or through the development of culturally competent guidelines for researchers. In the US, the National Institutes of Health Revitalization Act introduced in 1993 required all federally funded research to make genuine efforts to include women and minorities in research. Comparison of biobank and US census data shows a high degree of ethnic diversity in biobanks, with white, black and Native American people represented in accordance with their proportions in the overall population; Asian people were over‐represented. However, there was an under‐representation of Hispanic/Latino people, likely due to recruitment and consent materials being primarily in English.^13^ Despite this under‐representation, US biobanks represent population diversity for most ethnic groups, including the proportion of Native Americans.

In the United Kingdom, UK Biobank (https://www.ukbiobank.ac.uk/), a large population‐based biobank, has no policy enforcing the inclusion of minorities, instead establishing an ethnicity recruitment subgroup to drive minority participation.^14^ Thirty thousand volunteers from minority ethnic groups are being recruited out of the total 500 000 to be about proportional (6% non‐white) with the population data from the 2001 census. To alleviate concerns of low sample size, each ethnic group will comprise at least 3000 individuals to ensure reliable analysis between populations. This approach represents a compromise to address the needs of an ethnically diverse population and the concerns of researchers in producing statistically relevant data.

In Aotearoa/New Zealand, culturally informed guidelines were produced following a 3‐year research project.^15^ The Te Mata Ira genomic research and He Tangata Kei Tua biobanking guidelines draw upon mātauranga (Māori knowledge) and tikanga (Māori customs) to provide an ethical framework for genomic research and biobanking practice. This includes practical features such as having information and consent forms in Māori language, and the option to dispose of the samples in ceremony with appropriate karakia, a traditional form of prayer.^15^ This recognition of cultural values helps to both drive Māori engagement and assist researchers in implementing recommended practices.

## Diversity in Australian biobanks

In Australia, there is no specific legislative framework to guide the inclusion of Indigenous and other minority groups in biobanks or biomedical research. This is reflected in the low levels of Indigenous participation in Australian biobanks.

We contacted 21 Australian biobanks to take part in an online survey; of these, 16 returned fully completed responses. The biobanks ranged in size from 60 to 20 000 participants, with only 50% of biobanks recording whether the participant identified as Indigenous. When biobanks were asked to provide an approximation of participants who identified as Indigenous, 67% of respondents indicated 0–1%, with the remainder unsure (nationally, the Indigenous population is estimated to be 3.3%^16^).

The majority (81%) of biobanks believed engagement with local Indigenous communities was important, yet only 13% were aware of such engagement ever taking place. Engagement with local Indigenous communities was viewed as having positive outcomes for both the communities and biobanks by leading to greater participation by local Indigenous people, making the biobank more ethically sound, raising questions that the biobanks have not considered and establishing ongoing links with the communities. Despite this, a quarter of biobank respondents believed that a requirement to engage with Indigenous communities would add significantly to the difficulties in getting the biobank established, even considering numerous perceived benefits. They cited problems such as the lack of suitable personnel to be involved in community engagement, the necessity to establish suitable governance in line with community feedback, and increased difficulties in gaining ethics approval.

Interestingly, despite 20% of Australians speaking a language other than English at home, only 25% of biobanks surveyed had participant information sheets/consent forms that had been adapted for non‐English speakers and none had been adapted specifically for Indigenous participants. Without adapted written information and consent forms explaining what is expected of the participant, or the presence of an interpreter, informed consent cannot be given by non‐English speakers.

The results of our survey reflect a common problem. Despite recognition of the need for Indigenous representation, researchers are remiss in how to achieve this in light of their own operational challenges. With little progress in improving Indigenous inclusion in Australian biobanks, we must look at what has been successful internationally and how this could be incorporated into our own local practices.

## Where to next?

The relationship model for biobanks developed to address Māori ethical concerns in Aotearoa/New Zealand provides us with a successful framework on which to further develop Australian guidelines. This model is founded on principles of open communication and community respect. Successful aspects include:

■ improved communication and ongoing engagement at the individual and community level;
■ dynamic consent allowing for Māori participants to only take part in research that benefits their communities;
■ the ceding of ownership of samples with researchers acting as stewards; and
■ extensive provisions made for culture and tradition.

The heterogeneity of Indigenous people in terms of language, culture and customs makes comprehensive guidelines difficult to produce; however, these key aspects from the New Zealand model would be broadly applicable in the Australian context. This is possible through a community‐based, participant‐centric approach where Indigenous participants are key in the decision‐making process to ensure cultural sensitivity, while simultaneously safeguarding the relevance of the research to the communities.

In 2010, the National Health and Medical Research Council released a biobanks information paper which provided recommendations for the inclusion of Indigenous and other minority groups in Australian biobanks.^17^ While a step in the right direction, without guidelines to shape our dialogue or regulations to ensure that these recommendations are implemented, the inclusion of Indigenous people in biobanks remains stagnant. To further these aims, the National Centre for Indigenous Genomics is continuing to develop a model for research engagement and a dynamic consent platform for Indigenous Australians which will provide researchers with a valuable framework for ethical research and guidelines for culturally competent engagement.

It is unclear whether such guidelines would be widely adopted by biobanks without regulation, due to the operational and budget constraints identified in our survey. However, there is a clear need to continue the development of guidelines for the inclusion of Indigenous Australians in biobanks and to encourage their implementation. Success internationally has shown us that diversity in our biobanks can be improved by applying culturally competent practices and through active recruitment and community engagement. It has also shown us that without any direct action by Australian biobanks to improve Indigenous participation, the improved health outcomes promised by the genomic era will not be shared equitably.

## Competing interests

No relevant disclosures.

## Provenance

Not commissioned; externally peer reviewed.
